# Adverse Events and Unsuccessful Intubation Attempts Are Frequent During Neonatal Nasotracheal Intubations

**DOI:** 10.3389/fped.2021.675238

**Published:** 2021-05-11

**Authors:** Susanne Tippmann, Martin Haan, Julia Winter, Ann-Kathrin Mühler, Katharina Schmitz, Mascha Schönfeld, Luise Brado, Seyed Hamidreza Mahmoudpour, Eva Mildenberger, André Kidszun

**Affiliations:** ^1^Division of Neonatology, Department of Pediatrics, University Medical Center of the Johannes Gutenberg-University Mainz, Mainz, Germany; ^2^Division of Medical Biostatistics and Bioinformatics, Institute of Medical Biostatistics, Epidemiology and Informatics, University Medical Center of the Johannes Gutenberg-University Mainz, Mainz, Germany; ^3^Division of Neonatology, Department of Pediatrics, Inselspital, Bern University Hospital, University of Bern, Bern, Switzerland

**Keywords:** intubation, neonate, adverse event, intubation attempt, nasotracheal

## Abstract

**Background:** Intubation of neonates is difficult and hazardous. Factors associated with procedure-related adverse events and unsuccessful intubation attempts are insufficiently evaluated, especially during neonatal nasotracheal intubations.

**Objective:** Aim of this study was to determine the frequency of tracheal intubation–associated events (TIAEs) during neonatal nasotracheal intubations and to identify factors associated with TIAEs and unsuccessful intubation attempts in our neonatal unit.

**Methods:** This was a prospective, single-site, observational study from May 2017 to November 2019, performed at a tertiary care neonatal intensive care unit in a German academic teaching hospital. All endotracheal intubation encounters performed by the neonatal team were recorded.

**Results:** Two hundred and fifty-eight consecutive intubation encounters in 197 patients were analyzed. One hundred and forty-eight (57.4%) intubation encounters were associated with at least one TIAE. Intubation inexperience (<10 intubation encounters) (OR = 2.15; 95% CI, 1.257–3.685) and equipment problems (OR = 3.43; 95% CI, 1.12–10.52) were predictive of TIAEs. Intubation at first attempt (OR = 0.10; 95% CI, 0.06–0.19) and videolaryngoscopy (OR = 0.47; 96% CI, 0.25–0.860) were predictive of intubation encounters without TIAEs. The first intubation attempt was commonly done by pediatric residents (67.8%). A median of two attempts were performed until successful intubation. Restricted laryngoscopic view (OR = 3.07; 95% CI, 2.08–4.53; Cormack-Lehane grade 2 vs. grade 1), intubation by pediatric residents when compared to neonatologists (OR = 1.74; 95% CI, 1.265–2.41) and support by less experienced neonatal nurses (OR = 1.60; 95% CI, 1.04–2.46) were associated with unsuccessful intubation attempts.

**Conclusions:** In our unit, TIAEs and unsuccessful intubation attempts occurred frequently during neonatal nasotracheal intubations. To improve success rates, quality improvement und further research should target interprofessional education and training, equipment problems and videolaryngoscopy.

## Introduction

In neonates, endotracheal intubation and mechanical ventilation are avoided whenever possible to reduce damage of the immature lung (1). Nevertheless, endotracheal intubation is a necessary and frequently performed procedure. It can be estimated that endotracheal intubation occurs in around 20% of admissions to the neonatal intensive care unit (NICU). Endotracheal intubation of neonates is often lifesaving but also difficult to perform and hazardous. Depending on intubation experience, first-pass success rate is around 20–70% (2, 3). In many cases, three or more attempts are necessary until successful intubation (2, 4). Tracheal intubation–associated events (TIAEs) occur in up to 40% of neonatal intubations (4). Frequent TIAEs include esophageal intubation, airway injury, bleeding, desaturations, and bradycardias (2, 4). Previous studies suggest that intubation inexperience and multiple intubation attempts are predictive of TIAEs (2, 4–6). Only sparse data are available regarding factors associated with unsuccessful intubation attempts and trainers and trainees often disagree about the reasons of failed intubation attempts (7–9). There is an evident lack of knowledge on TIAEs and success rates in neonatal centers outside the US. This is of particular importance since many centers in Europe prefer nasotracheal to orotracheal intubation. Differences in personnel involved, different educational systems and procedural approaches might affect the efficacy and safety of neonatal intubations. Aim of this study was to determine the frequency of TIAEs during neonatal nasotracheal intubations and to identify factors associated with TIAEs and unsuccessful intubation attempts in our neonatal unit.

## Methods

### Setting and Procedures

This was a prospective observational study performed at a German tertiary care NICU from May 2017 to November 2019. All consecutive endotracheal intubation encounters performed by the neonatal team were recorded. That included intubation encounters in the delivery room (DR), in the NICU and during neonatal transport.

In our unit, intubations are performed by an interprofessional team, usually consisting of a neonatal nurse, a pediatric resident and a neonatologist. Nasotracheal intubation is commonly performed as a two-step procedure. First, the endotracheal tube (ETT) is carefully introduced 4–5 cm through the nose. Prior to inserting the laryngoscope, bag ventilation and pre-oxygenation is performed *via* the inserted ETT. Finally, the glottis is visualized *via* direct or videolaryngoscopy and the ETT is advanced through the vocal cords with the help of a Magill forceps. Correct insertion of the ETT is verified clinically (auscultation, adequate chest excursions) and subsequently via chest X-ray.

During the study period, no written standard operating procedure was available. Pediatric residents were usually performing the first intubation attempt. Although not specified, usually not more than 2-3 failed attempts were allowed until a more experienced physician took over. Two types of ETTs were used. Cuffless, transparent Vygon tubes with a lateral port for surfactant delivery were usually used in the delivery room, while cuffless, transparent, Mallinckrodt tubes without a lateral port were used for re-intubations in the NICU. If given, premedication consisted of a fentanyl, and/or diazepam, and vecuronium. Dose regimes were variable.

### Patients and Endpoints

All live born infants intubated by the neonatal team were included in the analysis. An intubation encounter was defined as the whole course from preparation to final ETT placement and verification of the correct tip position *via* chest x-ray. Each insertion of the laryngoscope into the oral cavity was defined as an intubation attempt.

The study's primary endpoint was the frequency of TIAEs. TIAEs encompassed: death, resuscitation, airway injury, hemorrhage, chest wall rigidity, emesis, esophageal intubation with or without concomitant desaturation, treatment of arterial hypotension, treatment of pain/discomfort, new occurrence or progression of intraventricular hemorrhage, pneumothorax, mainstem intubation, difficult bag mask ventilation, equipment failure, and transition from non-emergent to emergent intubation. Concomitant oxygen desaturations and bradycardias were recorded separately. Secondary endpoint was the analysis of factors associated with TIAEs and unsuccessful intubation attempts.

The local ethics committee of the Rhineland-Palatinate Medical Association approved this study. A waiver of informed consent was granted. Participants were not subjected to any study-related measures. The study is registered at the German clinical trial database DRKS (identifier: DRKS00013575).

### Data Collection, Sample Size Calculation and Data Analysis

Intubation encounters were documented by the physician in charge. Documentation was based on a previously published study and included individual patient data, reasons for intubation, clinical data before intubation, premedication, equipment, data on TIAEs, desaturations (SpO_2_ <80%; <60%), bradycardias [heart rate (HR) <100/min; <60/min], and characteristics of all individual intubation attempts including laryngoscopic view (4). Laryngoscopic view was graded according to Cormack-Lehane (Supplementary Figure 1). Following data pseudonymization, study personnel transferred the data into a digital database using a double-check approach.

The study was designed to last 24 months. It was expected that TIAEs would occur in 40% of intubations. The true relative frequency should be estimated with an accuracy of 10%. The power calculation was based on a two-sided 95% confidence interval with an approximate normal distribution. A minimum sample size of 93 patients was calculated.

We summarized the data by using descriptive statistics, frequencies and mean with standard deviations for the categorical and continuous variables, respectively. We examined the factors associated with procedure-related adverse events and unsuccessful intubation attempts using univariate logistic regression analysis. All statistical analyses were performed with SPSS software, version 23 (IBM SPSS, Armonk, NY, USA).

## Results

Two hundred and fifty-eight intubation encounters in 197 patients and 621 intubation attempts were recorded. Most intubation encounters occurred in the DR (52.7%). In 222/258 (86.0%) intubation encounters, infants were cardiorespiratory stable (SpO_2_ >90%, HR >100/min) prior to intubation. The main indications for endotracheal intubation were oxygenation failure, respiratory acidosis, and apnea. During the 2.5-year study period, 5/258 (1.9%) intubation encounters were performed *via* the orotracheal route. Only 4/258 (1.6%) intubation encounters were carried out due to unplanned extubations. In contrast to the NICU (94.7%), only a minority of infants received premedication in the DR (38.2%). The main reasons given for not providing premedication were classification as emergency in 16.4% and consideration as “not necessary” in another 16.0%. Premedication was not readily available in 2.0% of intubation encounters. Following chest X-ray, a correction of the initial tube insertion depth was done in 73/258 (28.3%) intubation encounters. The detailed characteristics of intubation encounters and procedural characteristics are presented in Tables 1, 2.

**Table 1:** Characteristics of intubation encounters.

	**NICU (*n* = 114)**	**DR (*n* = 136)**	**Transport (*n* = 8)**
Gestational age at birth, wk, median (IQR)	29 (25–35)	29 (26–33)	40 (38–41)
Birth weight, g, median (IQR)	1,127 (720–2,117)	1,220 (728–2,148)	3,325 (2,970–3,580)
Postmenstrual age at intubation, wk, median (IQR)	31 (27–36)	N/A^*^	N/A^*^
Weight at intubation [g] Median, IQR	1,363 (835–2,100)	N/A^*^	N/A^*^
Nasotracheal intubation, *n* (%)	113 (99.1)	132 (97.1)	8 (100)
Emergent intubation, *n* (%)	10 (8.8)	37 (27.2)	3 (37.5)
Intubation success rates, *n* (%)			
First attempt	48 (42.1)	50 (36.7)	2 (25.0)
Second attempt	24 (21.1)	32 (23.5)	2 (25.0)
Three or more attempts	42 (36.8)	54 (39.7)	4 (50.0)
Patient's condition before intubation^#^, *n* (%)			
FiO_2_ >0.21	100 (87.7)	130 (95.6)	8 (100)
CPAP	90 (78.9)	64 (47.1)	3 (37.5)
Bag-mask ventilation	32 (28.1)	104 (76.5)	7 (87.5)
Cardiac compressions	0	6 (4.4)	2 (25.0)
Cardiorespiratory stable (SaO_2_ >90%, HR >100/min), *n* (%)	109 (95.6)	108 (79.4)	5 (62.5)
Indication for intubation^#^, *n* (%)			
Oxygenation failure	77 (67.5)	119 (87.5)	8 (100)
Respiratory acidosis	39 (34.2)	61 (44.9)	7 (87.5)
Apneas	43 (37.7)	18 (13.2)	4 (50)
Resuscitation	2 (1.8)	15 (11)	2 (25)
Surfactant administration	28 (24.6)	89 (65.4)	1 (12.5)
Tachypnea/dyspnea	47 (41.2)	63 (46.3)	1 (12.5)
Upper airway obstruction	7 (6.1)	7 (5.1)	0
Elective (surgery, transport)	7 (6.1)	1 (0.7)	0
Unplanned extubation	3 (2.6)	1 (0.7)	0
Other (pneumothorax, abdominal distension, choanal atresia, ETT obstruction)	20 (17.5)	19 (14)	0

* *Infants were intubated at birth;*

# *More than one indication could be reported per patient; CPAP, continuous positive airway pressure; CPR, cardiopulmonary resuscitation; ETT, endotracheal tube; FiO_2_, fraction of inspired oxygen; HR, heart rate; N/A, not applicable; SaO_2_, oxygen saturation measured by pulse oximetry; SD, standard deviation*.

**Table 2:** Procedural characteristics of intubation encounters.

	**NICU intubations (*n* = 114)**	**DR intubations (*n* = 136)**	**Transport intubations (*n* = 8)**
Category			
Premedication, *n* (%)			
Diazepam only	2 (1.8)	25 (18.4)	1 (12.5)
Fentanyl only	2 (1.8)	5 (3.7)	0
Diazepam + fentanyl	34 (29.8)	14 (10.3)	4 (50.0)
Diazepam + fentanyl + Vecuronium	68 (59.6)	8 (5.9)	0
Any premedication	108 (94.7)	52 (38.2)	5 (62.5)
First device used, *n* (%)			
Direct laryngoscopy	86 (75.4)	108 (79.4)	8 (100)
Videolaryngoscopy	28 (24.6)	28 (20.6)	0
ETT used, *n* (%)			
Unknown	2 (1.8)	2 (1.5)	1 (12.5)
With surfactant bypass	38 (33.3)	95 (69.9)	5 (62.5)
Without surfactant bypass	74 (64.9)	39 (28.7)	2 (25.0)
First attempt proceduralist, *n* (%)			
Pediatric resident	84 (73.7)	84 (61.8)	7 (87.5)
Neonatologist	30 (26.3)	52 (38.2)	1 (12.5)
Physician's experience, *n* (%)			
<10 intubations	40 (35.1)	36 (26.5)	3 (37.5)
10–40 intubations	44 (38.6)	48 (35.3)	4 (50.0)
>40 intubations	30 (26.3)	52 (38.2)	1 (12.5)
Neonatal nurse's experience, *n* (%)			
Unknown	3 (2.6)	2 (1.5)	1 (12.5)
<10 intubations	26 (22.8)	16 (11.8)	1 (12.5)
10–40 intubations	27 (23.7)	26 (19.1)	2 (25.0)
>40 intubations	60 (52.6)	91 (66.9)	4 (50.0)

*DR, delivery room; ETT, endotracheal tube; NICU, neonatal intensive care unit*.

Overall, 148/258 (57.4%) intubation encounters were associated with at least one TIAE. Esophageal intubation, with (41/258; 15.9%) or without (34/258; 13.2%) concomitant desaturation, was by far the most common TIAE. Table 3 summarizes the frequency and characteristics of TIAEs in detail. Moderate desaturations (SpO_2_ <80%) and bradycardias (HR <100/min) were common and occurred during 45.9 and 25.7% of intubation encounters. Severe desaturations (SpO_2_ <60%) and bradycardias (HR <60/min) occurred during 17.1 and 6.2% of intubation encounters, respectively.

**Table 3:** Number and characteristics of observed TIAEs.

	**NICU intubations (*n* = 114)**	**DR intubations (*n* = 136)**	**Transport intubations (*n* = 8)**
Category of TIAE, *n* (%)^*^			
Death	0 (0)	1 (0.7)	0 (0)
Resuscitation	0 (0)	0 (0)	0 (0)
Airway injury	3 (2.6)	7 (5.1)	0 (0)
Hemorrhage	12 (10.5)	12 (8.8)	0 (0)
Chest wall rigidity	10 (8.8)	3 (2.2)	0 (0)
Emesis	0 (0)	0 (0)	0 (0)
Esophageal intubation			
Without desaturation	17 (14.9)	15 (11.0)	2 (25.0)
With desaturation	16 (14.0)	24 (17.6)	1 (12.5)
Treatment of arterial hypotension	7 (6.1)	18 (13.2)	3 (37.5)
Treatment of pain or discomfort following intubation	15 (13.2)	17 (12.5)	4 (50.0)
New occurrence or progression of intraventricular hemorrhage^#^	0 (0)	1 (0.7)	0 (0)
Pneumothorax	0 (0)	15 (11.0)	0 (0)
Mainstem intubation	4 (3.5)	2 (1.5)	1 (12.5)
Difficult bag-mask ventilation	11 (9.6)	15 (11.0)	0 (0)
Equipment failure	12 (10.5)	10 (7.4)	0 (0)
Turning to emergency	6 (5.3)	1 (0.7)	0 (0)
Others/unspecified	7 (6.1)	3 (2.2)	0 (0)

* *Some intubation encounters were associated with multiple TIAEs;*

# *Analysis only includes encounters with pre-intubation cranial imaging; TIAE, tracheal intubation-associated event; IE, intubation encounter; NICU, neonatal intensive care unit; DR, delivery room; CPR, cardiopulmonary resuscitation; IVH, intraventricular hemorrhage*.

Intubation inexperience (<10 intubation encounters) (OR = 2.15; 95% CI, 1.257–3.685) and equipment problems (OR = 3.43; 95% CI, 1.12–10.52) were predictive of TIAEs. Intubation at first attempt (OR = 0.10; 95% CI, 0.06–0.19) and videolaryngoscopy (OR = 0.47; 96% CI, 0.25–0.860) were associated with intubation encounters without TIAEs. Factors associated with the occurrence of any TIAE are summarized in Table 4.

**Table 4:** Factors associated with TIAEs.

**Condition**	**Unadjusted OR (95% CI)**
First attempt intubation	0.10 (0.06–0.19)
Premedication with vecuronium	1.33 (0.188–9.39)
Prior cardiorespiratory stabilization possible	0.46 (0.21–1.01)
Emergency intubation	1.26 (0.67–2.39)
Delivery room intubation	0.93 (0.57–1.53)
Intubation during on-call times (17:00–8:00)	1.15 (0.70–1.89)
Equipment problems	3.43 (1.12–10.52)
Birth weight <1,000 g	0.796 (0.48–1.31)
Videolaryngoscopy^*^	0.47 (0.25–0.860)
Inexperienced proceduralist^#^	2.15 (1.257–3.685)
Inexperienced nurse^#^	0.68 (0.347–1.36)

*CI, confidence interval; OR, odds ratio; TIAE, tracheal intubation-associated event.*

* *Videolaryngoscopy used at first attempt.*

# *Experience <10 intubations*.

A median of two attempts were performed until successful intubation. The first intubation attempt was most commonly done by pediatric residents (67.8%). Restricted laryngoscopic view (OR = 3.07; 95% CI, 2.08–4.53; Cormack-Lehane grade 2 compared to grade 1), intubation by pediatric residents when compared to neonatologists (OR = 1.74; 95% CI, 1.265–2.41) and less experienced neonatal nurses (OR = 1.60; 95% CI, 1.04–2.46) were associated with unsuccessful intubation attempts. Details on unsuccessful intubation attempts are given in Table 5.

**Table 5:** Factors associated with unsuccessful intubation attempts (*n* = 363) vs. successful attempts (*n* = 258).

**Condition**	**Unadjusted OR (95% CI)**	
Laryngoscopic view^*^	Grade 1	Ref
	Grade 2	3.07 (2.08–4.53)
	Grade 3	13.52 (6.87–26.60)
	Grade 4	N/A
Physicians' expertise	Neonatologist	Ref
	Pediatric resident	1.74 (1.26–2.41)
Physicians' experience^#^	More than 40	Ref
	Between 10 and 40	1.84 (1.27–2.67)
	<10	1.54 (0.99–2.38)
Nurses' expertise	Specialized nurse^+^	Ref
	Nurse	1.24 (0.90–1.71)
Nurses' experience^§^	More than 40	Ref
	Between 10 and 40	1.66 (1.11–2.48)
	<10	1.60 (1.04–2.46)
Gastric tube in place (*n* = 135)	Yes	Ref
	No	0.78 (0.53–1.16)
Videolaryngoscopy (*n* = 112)	Yes	Ref
	No	1.220 (0.80–1.84)

*CI, confidence interval; N/A, not applicable; OR, odds ratio; ref, reference.*

* *Grades refer to Cormack-Lehane, data available for 604/621 attempts.*

# *Number of intubations previously performed.*

+ *Specialized nurses have received structured, postgraduate training in neonatal intensive care nursing.*

§ *Number of intubations previously assisted, data available for 610/621 attempts*.

## Discussion

Here, we report characteristics of neonatal nasotracheal intubation encounters and factors associated with TIAEs and unsuccessful intubation attempts. In our unit, nasotracheal intubation encounters were frequently associated with TIAEs. The frequency of TIAEs (57.4%) observed in this study appears to be higher when compared to previous reports from orotracheal intubation encounters (2, 4). Foglia et al. and Hatch et al. reported rates of TIAEs basically between 20 and 40% (2, 4, 5). Three main factors might account for this difference. First, although not proven, one might argue that the nasotracheal intubation procedure itself is more difficult to learn and to perform. During nasotracheal intubation, besides the laryngoscope, an additional instrument, the Magill forceps, needs to be entered into the oral cavity and may make it more challenging to advance the ETT through the vocal cords. The nasotracheal approach may also last longer as the ETT needs to be introduced through the nose first. Second, levels of individual intubation expertise may vary significantly across studies and third, hospital staffing structures and specialist training systems differ between countries. In contrast to other reports, in our study, most first attempts were performed by pediatric residents, who commonly have little intubation experience (2).

Several studies have shown, that intubation inexperience was associated with TIAEs but also unsuccessful intubation attempts (10). Our study confirms the reports on orotracheal intubations showing that nasotracheal intubations at first attempt were associated with less TIAEs (4). First pass-success rates observed in our study are low but comparable to previously reported rates (2). In line with previous reports, we did observe only unspecific differences of TIAEs when comparing NICU and DR intubations (11).

First-pass success rates, frequency of TIAEs and desaturations and bradycardias are the hallmarks of intubation quality. When compared to older children and adults, neonatal intubations appear to be remarkably more difficult and associated with more TIAEs (12, 13). This does not necessarily arise from anatomy and physiology. Neonates have small airways and anatomic peculiarities when compared to adult airways, but they typically do not have difficult airways (14). It must be acknowledged that neonates that need to be intubated are often cardiorespiratory unstable. Only one study found a gestational age <32 weeks and a birth weight <1,500 g to be associated with difficult intubations (6). In our study, extremely low birth weight and emergent intubations were not associated with lower success rates and higher rates of TIAEs, respectively. We did observe a high rate of equipment failures as well as an association of equipment failures with TIAEs. Quality improvement initiatives may improve success rates and reduce TIAEs (15). The frequency of TIAEs was reduced in one center following implementation of an intubation checklist and a time-out procedure (16). In addition, quality improvement might not only improve immediate patient safety but also long-term outcomes. There is some evidence from retrospective analyzes that successful first attempt intubation is associated with less intra-ventricular hemorrhage (IVH) in very preterm infants (17). Others did not observe this association but found DR intubations to be associated with higher odds of death and severe neurological injury when compared to NICU intubations (18).

In our study, use videolaryngoscopy and intubation by experienced proceduralists were predictive of intubation encounters without TIAEs. Videolaryngoscopy may help to prevent intubation failure and may also improve proper initial tube insertion depth. It is conceivable that facilitating the visualization of the larynx reduces mechanical stress and tissue damage of the upper airway and so increases patient comfort during the intubation procedure. In retrospective analysis, videolaryngoscopy was associated with fewer intubation-related adverse events (19). In small children, beyond the neonatal period and in a preoperative setting, videolaryngoscopy has been shown to reduce complications and improve success rates in a large multicenter randomized controlled trial (RCT) (20). Adequately powered RCTs will be required to study the effects of videolaryngoscopy in the neonatal intensive care setting.

Videolaryngoscopy was not associated with successful intubation attempts in our study. Successful intubation attempts were associated with a good laryngoscopic view and more experienced interprofessional team members. Sufficient laryngoscopic view might be operator dependent, but our data suggest that training in laryngoscopy might improve intubation success and quality. Our study supports the idea that neonatal intubation is interprofessional teamwork. An important study result is the fact that physicians', but also neonatal nurses' experience appears to play a role in successful intubation. Neonatal intubation proves to be a challenging provider skill that needs to be learned and practiced. Training should be performed as a team procedure, which might not only improve individual skills but also procedural flows and a culture of mutual support. It is also likely that more research on neonatal intubations will improve staff awareness. Besides simulated interprofessional training, videolaryngoscopy might have the potential to facilitate training and education. When compared to traditional direct laryngoscopy, videolaryngoscopy basically offers an improved view on the larynx, which may facilitate passing the endotracheal tube through the vocal cords. While it is difficult to visualize the vocal chords *via* direct laryngoscopy, especially during emergencies like meconium aspiration, videolaryngoscopy enables supervisors to see the larynx as well (9). In other studies, videolaryngoscopy has been shown to increase first-pass success rates when instructing neonatal residents (21, 22). Nevertheless, it remains unclear, whether videolaryngoscopy improves success rates in neonatal clinical routine, especially when used by staff already experienced in direct laryngoscopy (23).

Scientific evidence is almost absent that one route of endotracheal intubation is preferable to the other (24). Different approaches in centers worldwide resemble historically grown preferences rather than strategies based on scientific evidence. Beliefs exist that nasotracheal intubation is more comfortable, and that ETT fixation is simpler and more secure with respect to unplanned extubations. On the other hand orotracheal intubation is supposed to be easier to perform (25). It is clearly beyond the scope of this study to shed new light on these questions, but we have observed a very low number of intubations due to unplanned extubations. In contrast, Hatch et al. have observed that in their study of orotracheal intubations unplanned extubations accounted for 62% of emergent intubations (4). We have also observed, that in some rare cases nasotracheal intubation is simply not possible (e.g., choanal atresia) while in other cases it was difficult to introduce the ETT through the nose.

We acknowledge the limitations of the study, which are basically its single-center and its observational design. Associations observed in this study do not allow conclusions on causalities but do generate hypotheses for further interventional studies. A particular strength of the study represents the complete recording of all consecutive intubation encounters and the detailed documentation of each particular intubation attempt.

In summary, in our unit, TIAEs and unsuccessful intubation attempts occurred frequently during neonatal nasotracheal intubations. To improve success rates, quality improvement und further research should target interprofessional education and training, equipment problems and videolaryngoscopy.

## Data Availability Statement

The original contributions presented in the study are included in the article/Supplementary Material, further inquiries can be directed to the corresponding author.

## Ethics Statement

The studies involving human participants were reviewed and approved by Ethics Committee of the Rhineland-Palatinate Medical Association, Deutschhausplatz 3, 55116 Mainz, Germany. Written informed consent from the participants' legal guardian/next of kin was not required to participate in this study in accordance with the national legislation and the institutional requirements.

## Author Contributions

ST: writing—original draft (lead), resources (supporting), formal analysis (supporting), and writing—review and editing (equal). MH: resources (lead), software (lead), and writing—review and editing (equal). JW and A-KM: resources (supporting), formal analysis (supporting), and writing—review and editing (equal). KS: formal analysis (supporting) and writing—review and editing (equal). MS: validation (supporting), formal analysis (supporting), and writing—review and editing (equal). LB: conceptualization (supporting), formal analysis (supporting), and writing—review and editing (equal). SM: formal analysis (lead) and writing—review and editing (equal). EM: supervision (lead), validation (lead), methodology (equal), conceptualization (equal), formal analysis (supporting), and writing—review and editing (equal). AK: methodology (lead), conceptualization (lead), writing—original draft (supporting), formal analysis (supporting), and writing—review and editing (lead). All authors approved the final manuscript as submitted and agree to be accountable for all aspects of the work.

## Conflict of Interest

The authors declare that the research was conducted in the absence of any commercial or financial relationships that could be construed as a potential conflict of interest.

## Supplementary Material

Supplementary Figure 1 Grading of laryngoscopic view according to Cormack-Lehane. Grade 1 = full view of the glottis, grade 2 = partial view of the glottis, grade 3 = epiglottis only visible, grade 4 = neither glottis nor epiglottis visible.
