# Supplementary figures and images for: Adverse Events and Unsuccessful Intubation Attempts Are Frequent During Neonatal Nasotracheal Intubations
Source: Front Pediatr. 2021 May 11;9:675238. doi: 10.3389/fped.2021.675238 (PMC8144442; doi:10.3389/fped.2021.675238)

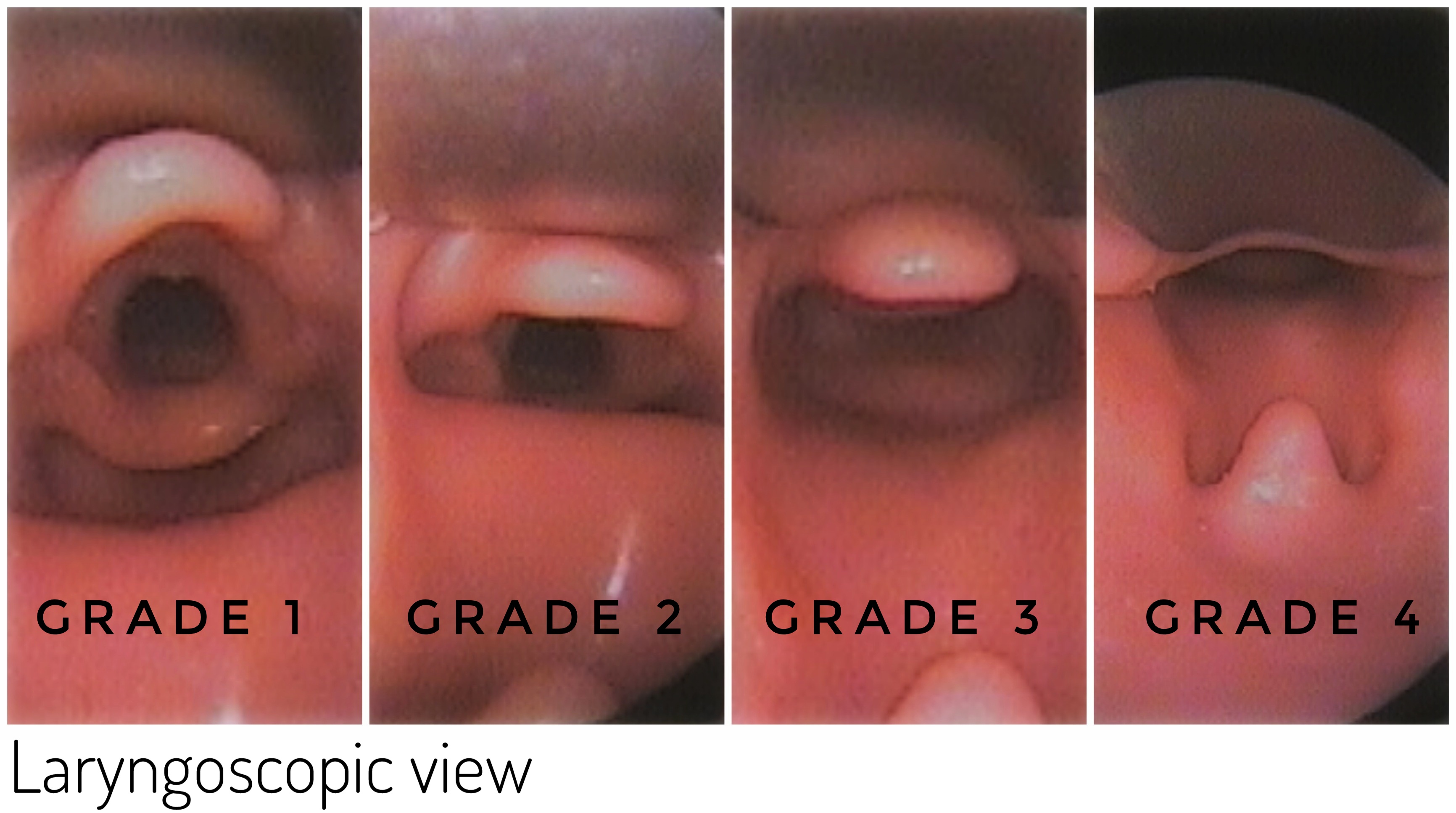

Supplement: Supplementary Figure 1 — Grading of laryngoscopic view according to Cormack-Lehane. Grade 1 = full view of the glottis, grade 2 = partial view of the glottis, grade 3 = epiglottis only visible, grade 4 = neither glottis nor epiglottis visible. [file Image_1.JPEG]
